# Quality of life in patients with strabismus: Assessment of the preoperative and postoperative psychosocial and functional aspects

**DOI:** 10.5935/0004-2749.2024-0118

**Published:** 2024-12-26

**Authors:** Ilse Elias Gomes Dorninger, David Leonardo Cruvinel Isaac, Alexandre Chater Taleb, Keila Monteiro de Carvalho, Marcos Pereira de Ávila

**Affiliations:** 1 Centro de Referência em Oftalmologia, Universidade Federal de Goiás, Goiânia, GO, Brazil; 2 Universidade Estadual de Campinas, Campinas, SP, Brazil

**Keywords:** Quality of life, Strabismus, Surveys and questionnaires, Psychosocial domain, Functional domain

## Abstract

**Purpose:**

To determine the influence of strabismus and its surgical correction on the preoperative and postoperative functional and psychosocial aspects of patients being treated at the CEROF/UFG Strabismus Outpatient Clinic.

**Methods:**

This prospective cross-sectional study included 27 patients, aged >7 years that were divided into two groups (<18 years and >18 years). The AS 20 questionnaire is composed of two domains (psychosocial and functional). Each domain includes 10 questions, which should be answered using a 5-point Likert scale. The questionnaire was administered preoperatively and 3 months postoperatively. In patients aged <18 years, the questionnaire was concurrently administered to their parents and/or guardians.

**Results:**

Preoperatively, the average psychosocial and functional scores were 55 (p=0.01) and 57.5 (p=0.025), respectively, in adults, 70 (p=0.03) and 78.7 (p=0.16), respectively, in children and adolescents, and 46.2 (p=0.002) and 57.5 (p=0.003), respectively, in the parents and/or guardians. Postoperatively, the average psychosocial and functional scores were 80 (p=0.01) and 82.5 (p=0.025), respectively, in adults, 81.2 (p=0.03) and 85 (p=0.16), respectively, in children and adolescents, and 83.7 (p=0.002) and 86.2 (p=0.003), respectively, in parents and/or guardians.

**Conclusion:**

The postoperative scores in the psychosocial (p=0.001), functional (p=0.001) and general (p<0.001) domains had increased in all the patients, demonstrating an improvement in the quality of life following strabismus correction surgery.

## INTRODUCTION

Strabismus is a defect in the positioning of one eye in relation to the other. Patients with this condition exhibit psychosocial impairment due to self-perception and perception by others and functional impairment due to sensory damage caused by ocular misalignment. These impairments may affect the quality of life of patients^(1^-^12)^.

The U-Report/SRSG-VAC survey was conducted by the United Nations International Emergency Fund in 2016^(13)^ regarding the experience of bullying. A total of 100,000 young people from 18 countries responded to the survey, and two-thirds of the respondents were victims of bullying. Of these victims, 25% were bullied for their physical appearance^(13)^. The United Na tions Global Study on Violence against Children, which was published in 2006^(14)^, revealed the negative effects of bullying on the physical and psychological health of children and adolescents: the more they are bullied, the more health problems that manifest (UN, 2006).

The adult strabismus-20 (AS-20) questionnaire (Supple-ment 1) was developed by Hatt et al. in English^(1)^. The AS-20 has been used in several countries and diffe-rent languages to evaluate adults and children with strabismus. This is a strabismus-specific questionnaire with 20 items that are distributed into the following two distinct subscales: psychosocial and functional. This questionnaire was translated into Portuguese and validated in Brazil by Margotto et al.^(2)^.

In this study we aimed to evaluate the psychosocial (related to social interaction) and functional (related to visual function) effects of strabismus on the lives of patients and the possible benefits of surgical treatment on the patients quality of life.

## METHODS

This prospective cross-sectional study was conducted at the Strabismus Outpatient Clinic of the Reference Center for Ophthalmology at the Federal University of Goiás (CEROF/UFG). It was conducted from October 2019 to February 2020 and from October 2020 to August 2021.

Patients aged ≥7 years of any sex, race or socioeconomic status, who were being monitored at the CEROF strabismus outpatient clinic and who had undergone strabismus correction surgery, following the SUS (Brazilian public healthcare system) queue, during the study period were included in the study.

The study included patients with horizontal deviations ≥20 prismatic diopters (DP), who underwent either a first surgery or reoperation at CEROF/UFG. Adults, adolescents and children with impaired cognitive capacity and adolescents and children of parents/guardians with impaired cognitive capacity were excluded from the study.

The Portuguese version of the AS 20 was used in this study^(2)^. The AS 20 is strabismus-specific set of 20 questions that is used to assess the health-related quality of life (HRQoL) of patients^(1)^. It comprises two different subscales (psychosocial and functional), and each question has five response options (never, rarely, sometimes, often or always [5-point Likert scale]). Each question is scored from 0 to 100 (0-always, 25-often, 50-sometimes, 75-rarely, and 100-never), with 100 points indicating the best quality of life. The final score is obtained by adding up the points for each question and dividing it by the number of questions in each subscale as well as the overall number of questions.

The same questionnaire was administered at two different times: preoperatively and at the third postoperative month. For the assessment of adolescent and children, the AS-20 was administered to the patients as well as their parents/guardians.

The anamnesis of all the patients was collected at the beginning of the study, and they all underwent a complete ophthalmological examination.

To evaluate the responses, the questions and variables studied were recorded in Excel under the psychosocial (question 1-10), functional (question 11-20), and general (all 20 questions) scales/domains. The average score of each domain was recorded. Furthermore, each question was evaluated individually, with its respective score.

All data were statistically analyzed using Statistical Package for the Social Sciences (SPSS) for Windows (version 21.0; SPSS Inc., Chicago, IL, USA).

The qualitative variables are presented as absolutes and relative frequencies. The association between the type of horizontal deviation (esotropia and exotropia) and presence or absence of amblyopia or visual impairment (VI) was analyzed using Fisher’s exact test.

The normality of quantitative variables was analyzed using the Kolmogorov-Smirnov and Shapiro-Wilk tests. In both tests, variables with a p-value of >0.05 were considered normally distributed.

The variation in the preoperative and postoperative questionnaire scores was analyzed by calculating the Δ value, which is obtained by subtracting the preoperative score from the postoperative score. The quantitative variables are presented as medians with minimum and maximum values.

The distribution of non-parametric quantitative variables (life questionnaire scores between two groups) was compared using the Mann-Whitney test.

The following groups were used for the comparisons: sex (male and female); presence or absence of amblyopia or low visual acuity; type of surgery (first or reoperation); type of horizontal deviation (esotropia or exotropia); magnitude of the deviation (medium angle [20-40 SD] or large angle [>40]); and association of horizontal deviation with vertical deviation (yes or no).

To compare the mean of two paired groups, such as the preoperative and postoperative quality of life scores, the Wilcoxon test was used for quantitative variables.

In all analyses, a significance level of 5% (p≤0.05) was adopted.

## RESULTS

Twenty-seven individuals, with a mean age of 20.89 ± 14.16 years and a median age of 16 years (range, 8-55), were evaluated. Table 1 includes data regarding the patient characteristics, such as sex, age group, type of horizontal deviation, type of surgery (first or reoperation), presence of amblyopia or VI, surgical success, magnitude of deviation (medium or large angle), and the presence or absence of vertical deviation in association with horizontal deviation.

**Table 1:** General characteristics of the study population

Variable	n (%)
**Sex**	
Female	15 (55.6)
Male	12 (44.4)
**Age**	
Child/adolescent	14 (51.9)
Adult	13 (48.1)
**Horizontal deviation**	
Esotropia	15 (55.6)
Exotropia	12 (44.4)
**Surgery**	
First	25 (92.6)
Reoperation	2 (7.4)
**Amblyopia or BAV**	
Yes	9 (33.3)
No	18 (66.7)
**Surgical success**	
Yes	26 (96.3)
No	1 (3.7)
**Deviation magnitude**	
20-40	2 (7.4)
>40	25 (92.5)
**Vertical deviation associated with horizontal**	
Yes	15 (55.6)
No	12 (44.4)

n= absolute frequency; %= relative frequency; BAV= low visual acuity.

Compared to the preoperative scores, the postoperative scores in the psychosocial (57.5 vs. 80.0, p=0.001), functional (75.0 vs. 85.0, p=0.001) and general (63.7 vs. 83.75, p<0.001) domains increased in all the study participants (Table 2). Furthermore, a comparison of the preoperative and postoperative scores in adults (Table 3), children and adolescents (Table 4), and guardians of the children and adolescents (Table 4) demonstrated an increase in all three domains (p<0.05).

**Table 2:** Comparison of the preoperative and postoperative questionnaire scores of all the study participants

Domain	Preoperative scoren=27	Postoperative scoren=27	p-value
**Psychosocial**			
Sum	575 [100-900]	800 [200-1,000]	0.001
Mean	57.5 [10.0-90.0]	80.0 [20.0-100.0]	0.001
**Functional**			
Sum	750 [300-925]	850 [150-1,000]	0.001
Mean	75.0 [30.0-92.5]	85.0 [15.0-100.0]	0.001
**General score**			
Sum	1,275 [525-1,775]	1,675 [575-2000]	<0.001
Mean	63.7 [26.3-88.8]	83.75 [28.8-100.0]	<0.001

Statistical test: Wilcoxon test.

**Table 3:** Comparison of the preoperative and postoperative questionnaire scores of the adult study population

Domain	Preoperative n=13	Postoperative n=13	p-value
**Psychosocial**			
Sum	550 [225-800]	800 [200-950]	0.010
Mean	55.0 [22.5-80.0]	80.0 [20.0-95.0]	0.010
**Functional**			
Sum	575 [300-925]	825 [150-975]	0.025
Mean	57.5 [30.0-92.5]	82.5 [15.0-97.5]	0.025
**General score**			
Sum	1,175 [525-1,550]	1,700 [575-1,875]	0.005
Mean	58.7 [26.3-77.5]	85.0 [28.8-93.8]	0.005

Statistical test: Wilcoxon test.

**Table 4:** Comparison of the preoperative and postoperative questionnaire scores of children and adolescents and their respective parents and/or guardians

Domain	Preoperative n=14	Postoperative n=14	p-value
**Children and adolescents**			
**Psychosocial**			
Sum	700 [100-900]	850 [625-1,000]	0.030
Mean	70.0 [10.0-90.0]	85.0 [62.5-100.0]	0.030
**Functional**			
Sum	800 [325-900]	850 [775-1,000]	0.016
Mean	80.0 [32.5-90.0]	85.0 [77.5-100.0]	0.016
**General score**			
Sum	1,425 [750-1,775]	1,650 [1,475-2000]	0.012
Mean	71.2 [37.5-88.8]	82.5 [73.8-100.0]	0.012
**Parents and/or guardians**			
**Psychosocial**			
Sum	475 [150-775]	850 [425-1,000]	0.002
Mean	47.5 [15.0-77.5]	85.0 [42.5-100.0]	0.002
**Functional**			
Sum	575 [350-950]	875 [600-1,000]	0.003
Mean	57.5 [35.0-95.0]	87.5 [60.0-100.0]	0.003
**General score**			
Sum	1,025 [700-1,575]	1,650 [1,025-2000]	0.001
Mean	51.2 [35.0-78.8]	81.9 [51.3-100.0]	0.001

Statistical test: Wilcoxon test.

There was a significantly higher (p=0.041) preoperative functional domain score in the group without amblyopia or VI than in the group with amblyopia or VI. Furthermore, there was no statistically significant difference in the preoperative and postoperative score between patients undergoing the first surgery and those undergoing reoperation. However, the variation was statistically significantly greater variation in patients undergoing the first surgery than in those undergoing reoperation.

There was no significant difference in the preoperative and postoperative psychosocial domain scores between patients with esotropia and those with exotropia. The preoperative functional and general domain scores were significantly higher among patients with esotropia than among those with exotropia. However, there was no significant difference in the postoperative scores.

Furthermore, the variation in scores was significantly greater patients with exotropia than in patients with esotropia.

Only one patient with esotropia (6.7%) had amblyopia and/or VI. However, eight patients with exotropia (66.7%) had amblyopia and/or VI. Overall, nine patients with a horizontal deviation (33.3%) had amblyopia or VI. Therefore, there was a statistically significant association between the type of horizontal deviation and the presence of amblyopia and/or VI (p=0.003).

There was no statistically significant difference in the preoperative scores and variation in the preoperative and postoperative scores between patients in the different angle groups and between patients with and without vertical deviation in association with horizontal deviation.

When evaluating each question individually, there were significantly more question that were scored significantly higher postoperatively by parents and/or guardians (Table 7) than by the adults (Table 5) or children and adolescents (Table 6).

**Table 5:** Comparison of the preoperative and postoperative scores of individual question in the adult study population

Question	Preoperative n=13	Postoperative n=13	p-value
Q1	50 [0-100]	75 [0-100]	0.014
Q2	50 [0-100]	50 [0-100]	0.293
Q3	25 0 [0-100]	75 [0-100]	0.065
Q4	50 [0-100]	75 [25-100]	0.038
Q5	75 [0-100]	100 [50-100]	0.068
Q6	0 [0-100]	0 [0-100]	0.490
Q7	75 [50-100]	100 [50-100]	0.102
Q8	75 [0-100]	100 [0-100]	0.062
Q9	50 [25-100]	100 [50-100]	0.006
Q10	50 [0-100]	100 [0-100]	0.164
Q11	75 [0-100]	100 [0-100]	0.161
Q12	100 [0-100]	100 [0-100]	0.705
Q13	75 [50-100]	100 [0-100]	0.408
Q14	50 [0-100]	100 [0-100]	0.056
Q15	50 [0-100]	75 [50-100]	0.128
Q16	75 [0-100]	75 [50-100]	0.200
Q17	50 [0-100]	100 [0-100]	0.065
Q18	0 [0-100]	50 [0-100]	0.150
Q19	75 [0-100]	100 [0-100]	0.088
Q20	75 [0-100]	100 [0-100]	0.150

Statistical test: Wilcoxon test.

**Table 6:** Comparison of the preoperative and postoperative scores of individual questions in the children and adolescents study population

Question	Preoperative n=14	Postoperative n=14	p-value
Q1	50 [0-100]	100 [0-100]	0.209
Q2	50 [0-100]	100 [50-100]	0.004
Q3	25 [0-100]	100 [50-100]	0.004
Q4	50 [0-100]	100 [50-100]	0.057
Q5	100 [0-100]	100 [0-100]	0.713
Q6	0 [0-100]	25 [0-100]	0.286
Q7	100 [25-100]	100 [50-100]	0.167
Q8	100 [0-100]	100 [100-100]	0.026
Q9	100 [25-100]	100 [0-100]	0.168
Q10	100 [0-100]	100 [50-100]	0.450
Q11	50 [0-100]	100 [50-100]	0.017
Q12	100 [0-100]	100 [75-100]	0.414
Q13	100 [50-100]	100 [75-100]	0.317
Q14	100 [25-100]	100 [50-100]	0.730
Q15	75 [50-100]	75 [50-100]	1.000
Q16	100 [0-100]	100 [75-100]	0.059
Q17	75 [0-100]	100 [50-100]	0,062
Q18	25 [0-50]	75 [0-100]	0,022
Q19	100 [0-100]	100 [75-100]	0,042
Q20	100 [0-100]	100 [50-100]	0,071

Statistical test: Wilcoxon test.

**Table 7:** Comparison of the preoperative and postoperative scores of individual questions in the parents/guardians study group

Question	Preoperative n=14	Postoperative n=14	p-value
Q1	25 [0-100]	100 [0-100]	0.039
Q2	25 [0-100]	75 [0-100]	0.027
Q3	0 [0-50]	100 [0-100]	0.002
Q4	50 [0-100]	100 [0-100]	0.007
Q5	50 [25-100]	100 [50-100]	0.010
Q6	0 [0-50]	0 [0-100]	0.117
Q7	75 [25-100]	100 [0-100]	0.389
Q8	50 [0-100]	100 [50-100]	0.017
Q9	50 [0-100]	75 [50-100]	0.034
Q10	50 [0-100]	100 [50-100]	0.027
Q11	100 [25-100]	100 [50-100]	0.252
Q12	75 [50-100]	100 [75-100]	0.021
Q13	75 [0-100]	100 [75-100]	0.025
Q14	75 [0-100]	100 [50-100]	0.016
Q15	50 [0-75]	75 [0-100]	0.007
Q16	75 [0-100]	100 [50-100]	0.014
Q17	50 [0-100]	100 [50-100]	0.055
Q18	0 [0-100]	25 [0-100]	0.254
Q19	75 [25-100]	100 [50-100]	0.047
Q20	50 [0-100]	100 [50-100]	0.013

Statistical test: Wilcoxon test.

Furthermore, the preoperative scores for questions 1, 10 and 15, and the postoperative score for question 4 was significantly lower in females than in males. However, there was no statistically significant difference in the overall domain score between the females and males (Tables 6 and 7).

The preoperative scores of questions 16, 17 and 19 were significantly lower in patients with exotropia type than in patients with esotropia. Furthermore, the preoperative scores of questions 14, 16 and 19 were significantly lower in patients with amblyopia or VI than in those without amblyopia or VI.

## DISCUSSION

The present study included 27 patients with strabismus. Of the 27 patients, 55.6% were female and 55.6% exhibited esotropia-type strabismus. These results are consistent with those of the study by Shimauti et al. that was conducted in Brazil^(3)^.

The patients were included at random according to the surgical queue order because CEROF is a public ophthalmology teaching institution. Furthermore, the services are provided via the SUS. During the study period, there was a shortage of supplies required for anesthetic procedures (such as muscle relaxants for orotracheal intubation) and the physical stricture of the surgical center was renovated due to the COVID-19 pandemic. Thus, the number of surgical procedures for paralytic and restrictive deviations as well as other more extensive and complex surgeries were limited. Most of the study participants had large-angle deviations (esotropia and exotropia), which may be attributed to the fact that CEROF is a referral center for ophthalmological treatments for the entire central-west and northern regions of the country.

As in the study by Amitava et al.^(4)^, in which patients with strabismus were evaluated before correction surgery and 3 months after it, our study did not identify a significant difference between the preoperative and postoperative scores in females and males. Furthermore, we found a statistically significant difference in only the preoperative functional score between patients with and those without amblyopia and/or low visual acuity. In our study, patients with amblyopia and/or VI (33.3% of the study population) exhibited lower scores than patients without amblyopia and/or VI. In the study by Amitava et al.^(4)^, this difference was mainly observed in the functional domain, which was consequently reflected in the general analysis. Furthermore, 40% of their study population had amblyopia.

In our study, there was no statistically significant difference in the preoperative and postoperative psychosocial domain score between patients with esotropia and those with exotropia. However, the preoperative functional and general domain score were statistically significantly higher among patients with esotropia than among patients with exotropia. Furthermore, there was no statistically significant difference in the postoperative scores.

In the study by Glasman et al.^(5)^, a total of 86 patients with strabismus were evaluated. Of the 86 patient, 60% had exotropia and 40% esotropia. We did not find a statistically significant difference in relation to the deviation direction, which is consistent with the finding of the study by Amitava et al.^(4)^.

In the present study, only one patient (6.7%) with esotropia and eight patients (66.7%) with exotropia exhibited amblyopia and/or VI. Furthermore, the preo-perative functional score was statistically significantly among patients without amblyopia and/or VI than among patients with amblyopia and/or VI. This may account for the higher functional score, which affects the general score, in patients with esotropia in this study.

We also found a statistically significant greater variation in the scores of all the evaluated items in patients with exotropia than in patients with esotropia. This may be attributed to the lower preoperative score in patients with exotropia (75.5 x 50).

In our study, there was no statistically significant difference in the preoperative and postoperative scores between patients undergoing their first surgery and those undergoing reoperation. This finding is consistent with that of the study by Hatt et al.^(1)^, which had a substantially more representative sample than that of the present study (n=46 vs. 2).

In the present study, the variation in preoperative and postoperative scores was there was a statistically significantly greater among patients undergoing the first surgery than among patients undergoing reoperation. In the study by Glasman et al.^(5)^, there was a strong correlation between deviations of greater magnitude and lower AS-20 scores. Greater deviations in patients undergoing the first surgery may account for the difference in variation. However, there was no statistically significant difference in preoperative scores and the variation in scores between patients with medium-angle deviations and those with large-angle deviations.

When evaluating each question individually, a significantly greater number of questions with a statistically significant increase in scores were observed in the group of parents and/or guardians than in the adult patients and adolescents or children. Furthermore, there were statistically significant differences in the score of some questions.

The preoperative score of questions 1, 10 and 15 and the postoperative score of question 4 was lower in females than in males. However, there were no statistically significant differences in the domain scores between females and males.

Furthermore, the preoperative scores of questions 16, 17 and 19, which are part of the functional domain, were lower in patients with exotropia than in patients with esotropia. The scores for questions 14, 16 and 19, which are part of the functional scale, were lower in patients with amblyopia or VI than in patients with amblyopia or VI.

In our study, the average preoperative scores of children and adolescents were much higher than the average scores of the adults and parents and/or guardians of the children. The mean postoperative scores were similar between the three groups. The preoperative findings were consistent with the findings of a study by Eiser et al.^(7)^. Eiser et al. conducted a systematic review of 14 studies to determine the relationship between assessments of children’s HRQoL made by themselves and those made by their parents^(7)^. There was greater evidence of agreement in the responses related to physical and functional aspects than in the responses related to emotional and social aspects. Furthermore, the parents’ responses predominantly indicated a poorer quality of life than those of the children. The study concluded that whenever possible, it is best to obtain information from children and parents.

We found an increase in the postoperative scores in the psychosocial (p=0.001), functional (p=0.001) and general (p<0.001) domains in all the study patients, indicating an improvement in the quality of life following the surgical correction of strabismus. These results are consistent with those of other studies that used the AS-20 questionnaire^(1^,^4^-^6^,^8)^ or other HRQoL assessment instruments in patients undergoing surgical correction of strabismus^(9^-^12)^.

The preoperative general score of the entire study population was 63.7. When stratified into adults, children and adolescents, and parents/guardians, the preoperative scores were 58.7, 71.2, and 51.2, respectively. The individual group scores and the overall score of 56 were lower (poorer quality of life) than that of visually normal adults (95; p<0.001) and patients with other eye diseases (86; p<0.001) in the study by Hatt et al.^(1)^.

We also found that the postoperative scores of the psychosocial (p=0.001), functional (p=0.001) and general (p<0.001) domains had increased in the entire population, demonstrating an improvement in the quality of life following the surgical correction of strabismus.

This study had some limitations, including a small size and statistical analyses using p as the only parameter. Future studies with a larger sample and/or other statistical analysis parameters could make the results more robust.

## Appendix

**Table:**

Supplement 1
**AS-20 Questionnaire**
The AS-20 is a self-administered questionnaire. At each follow-up visit, the questionnaire must be completed by the patient prior to the examination, unless otherwise instructed. Participants should be provided the instruction sheet and asked to review the instructions prior to completing the questionnaire. Responses are to be based on patient experience over the past month. All questions should be completed.
Instructions for patient
The AS-20 is a short questionnaire with statements about how strabismus (misaligned eyes) may affect you in your everyday life.
If you are unable to complete this on your own, please ask someone to assist you.
Instructions:
Please respond to EACH statement by circling the response that best reflects how you feel.
Circle only ONE response for each statement.
Please answer based on your experiences during the past month or since your last appointment if sooner.
If you wear glasses or contact lenses, respond as if you were wearing them unless otherwise instructed.
If you are not sure how to respond, please circle the response you think is most appropriate and make a comment in the margin.
If you have any questions please ask.
Thank you for completing this questionnaire.
Adult Strabismus Quality of Life Questionnaire (AS-20)
1) I worry about what people will think about my eyes.
( ) Never ( ) Rarely ( ) Sometimes ( ) Often ( ) Always
2) I feel that people are thinking about my eyes even when they do not say anything.
( ) Never ( ) Rarely ( ) Sometimes ( ) Often ( ) Always
3) I feel uncomfortable when people are looking at me because of my eyes.
( ) Never ( ) Rarely ( ) Sometimes ( ) Often ( ) Always
4) I wonder what people are thinking when they are looking at me because of my eyes.
( ) Never ( ) Rarely ( ) Sometimes ( ) Often ( ) Always
5) People do not give me opportunities because of my eyes.
( ) Never ( ) Rarely ( ) Sometimes ( ) Often ( ) Always
6) I am self-conscious about my eyes.
( ) Never ( ) Rarely ( ) Sometimes ( ) Often ( ) Always
7) People avoid looking at me because of my eyes.
( ) Never ( ) Rarely ( ) Sometimes ( ) Often ( ) Always
8) I feel inferior to others because of my eyes
( ) Never ( ) Rarely ( ) Sometimes ( ) Often ( ) Always
9) People react differently to me because of my eyes
( ) Never ( ) Rarely ( ) Sometimes ( ) Often ( ) Always
10) I find it hard to initiate contact with people I do not know because of my eyes
( ) Never ( ) Rarely ( ) Sometimes ( ) Often ( ) Always
11) I cover or close one eye to see things better
( ) Never ( ) Rarely ( ) Sometimes ( ) Often ( ) Always
12) I avoid reading because of my eyes
( ) Never ( ) Rarely ( ) Sometimes ( ) Often ( ) Always
13) I stop doing things because my eyes make it difficult to concentrate
( ) Never ( ) Rarely ( ) Sometimes ( ) Often ( ) Always
14) I have problems with depth perception
( ) Never ( ) Rarely ( ) Sometimes ( ) Often ( ) Always
15) My eyes feel strained
( ) Never ( ) Rarely ( ) Sometimes ( ) Often ( ) Always
16) I have issues reading because of my eye condition
( ) Never ( ) Rarely ( ) Sometimes ( ) Often ( ) Always
17) I feel stressed because of my eyes
( ) Never ( ) Rarely ( ) Sometimes ( ) Often ( ) Always
18) I worry about my eyes
( ) Never ( ) Rarely ( ) Sometimes ( ) Often ( ) Always
19) I cannot enjoy my hobbies because of my eyes
( ) Never ( ) Rarely ( ) Sometimes ( ) Often ( ) Always
20) I need to take frequent breaks when reading because of my eyes
( ) Never ( ) Rarely ( ) Sometimes ( ) Often ( ) Always

**Table:**

Supplement 1
**Questionário AS 20**
Instruções para o (a) paciente:
Este pequeno questionário, com 20 itens, contém declarações sobre como o estrabismo pode afetá-lo em seu dia-a-dia.
Se você for incapaz de preencher este questionário sozinho ou tiver qualquer dúvida, por favor pergunte a um dos pesquisadores.
Responda cada item fazendo um “X” na resposta que melhor reflete como você se sente.
Marque somente UMA resposta para cada item.
Se você usa óculos ou lente de contato responda os itens considerando o seu uso.
Se você não tem certeza da resposta, por favor, marque a resposta que é mais apropriada e faça um comentário na margem.
Quando o questionário fala em ”condições do olho” está se referindo a ESTRABISMO.
Obrigada por preencher o questionário.
Escala psicossocial:
1. Eu me preocupo com o que as pessoas vão pensar sobre os meus olhos.
( ) Nunca ( ) Raramente ( ) Às vezes ( ) Frequentemente ( ) Sempre
2. Eu sinto que as pessoas estão observando os meus olhos mesmo quando elas não dizem nada.
( ) Nunca ( ) Raramente ( ) Às vezes ( ) Frequentemente ( ) Sempre
3. Eu me sinto desconfortável quando as pessoas estão olhando para mim por causa dos meus olhos.
( ) Nunca ( ) Raramente ( ) Às vezes ( ) Frequentemente ( ) Sempre
4. Eu me pergunto o que as pessoas estão pensando quando estão olhando para mim por causa dos meus olhos.
( ) Nunca ( ) Raramente ( ) Às vezes ( ) Frequentemente ( ) Sempre
5. Eu sinto que as pessoas não me dão oportunidades por causa dos meus olhos.
( ) Nunca ( ) Raramente ( ) Às vezes ( ) Frequentemente ( ) Sempre
6. Eu tenho consciência sobre os meus olhos.
( ) Nunca ( ) Raramente ( ) Às vezes ( ) Frequentemente ( ) Sempre
7. As pessoas evitam olhar para mim por causa da condição dos meus olhos.
( ) Nunca ( ) Raramente( ) Às vezes ( ) Frequentemente ( ) Sempre
8. Eu me sinto inferior aos outros por causa da condição dos meus olhos.
( ) Nunca ( ) Raramente ( ) Às vezes ( ) Frequentemente ( ) Sempre
9. As pessoas reagem de maneira diferente comigo por causa da condição dos meus olhos.
( ) Nunca ( ) Raramente ( ) Às vezes ( ) Frequentemente ( ) Sempre
10. Acho difícil iniciar contato com pessoas que não conheço por causa da condição dos meus olhos.
( ) Nunca ( ) Raramente ( ) Às vezes ( ) Frequentemente ( ) Sempre
Escala funcional:
11. Eu cubro ou fecho um olho para ver melhor.
( ) Nunca ( ) Raramente ( ) Às vezes ( ) Frequentemente ( ) Sempre
12. Eu evito ler por causa dos meus olhos.
( ) Nunca ( ) Raramente ( ) Às vezes ( ) Frequentemente ( ) Sempre
13. Paro de fazer coisas porque meus olhos dificultam a concentração.
( ) Nunca ( ) Raramente ( ) Às vezes ( ) Frequentemente ( ) Sempre
14. Eu tenho problemas com a visão de profundidade.
( ) Nunca ( ) Raramente ( ) Às vezes ( ) Frequentemente ( ) Sempre
15. Sinto meus olhos cansados.
( ) Nunca ( ) Raramente ( ) Às vezes ( ) Frequentemente ( ) Sempre
16. Eu tenho problemas de leitura por causa da condição dos meus olhos.
( ) Nunca ( ) Raramente ( ) Às vezes ( ) Frequentemente ( ) Sempre
17. Eu me sinto estressado por causa da condição dos meus olhos.
( ) Nunca ( ) Raramente ( ) Às vezes ( ) Frequentemente ( ) Sempre
18. Eu me preocupo com a condição dos meus olhos.
( ) Nunca ( ) Raramente ( ) Às vezes ( ) Frequentemente ( ) Sempre
19. Eu não consigo aproveitar as coisas que gosto de fazer por causa dos meus olhos.
( ) Nunca ( ) Raramente ( ) Às vezes ( ) Frequentemente ( ) Sempre
20. Eu preciso fazer pausas frequentes quando leio por causa dos meus olhos.
( ) Nunca ( ) Raramente ( ) Às vezes ( ) Frequentemente ( ) Sempre
